# Acute Pancreatitis Secondary to Dengue Fever: An Uncommon Presentation of a Common Endemic Illness

**DOI:** 10.1155/2022/9540705

**Published:** 2022-12-13

**Authors:** Miguel A. Flor, Jéssica V. Andrade, Jorge A. Bucaram

**Affiliations:** ^1^Department of Internal Medicine, Luis Vernaza Hospital, Guayaquil, Ecuador; ^2^Universidad de Especialidades Espiritu Santo, Guayaquil, Ecuador; ^3^Instituto de Investigación e Innovación en Salud Integral, Universidad Católica de Santiago de Guayaquil, Guayaquil, Ecuador

## Abstract

Dengue viral infection is considered endemic in Ecuador. It is more frequent during winter, caused by an RNA virus in the Flavivirus group. Its presentation can range from an asymptomatic state to hemorrhagic fever with shock signs. Acute pancreatitis could be a rare form of acute abdomen presentation associated with dengue virus infection. This case illustrates a 26-year-old man who presents to the hospital with cramp-like pain in the epigastrium and radiation to the right upper quadrant, accompanied by nausea and vomiting. He also endorsed additional symptoms such as throbbing-like headache, myoarthralgias, and fever of 40.4°C (104.72°F). Laboratory tests revealed elevated hematocrit, thrombocytopenia, elevated pancreatic enzymes, transaminitis, elevated alkaline phosphatase (ALP), and gamma-glutamyl transferase (GGT). Ultrasonography of the abdomen revealed hepatic steatosis, free fluid in the abdominal cavity, and small bilateral pleural effusions. Additional testing revealed IgM and IgG antibodies positivity to dengue virus. The patient was treated conservatively with intravenous (IV) fluid hydration and bowel rest. Acute pancreatitis should be considered when a patient presents with a suspected acute abdomen in the emergency department, and a detailed medical history is necessary to make a correct approach to the differential diagnosis.

## 1. Introduction

Dengue is an infectious disease transmitted by mosquito vectors such as Aedes aegypti, caused by an RNA virus belonging to the Flavivirus group, which usually presents as a febrile illness called classic dengue [1].

There are four serotypes, DENV-1, DENV-2, DENV-3, and DENV-4; the frequency of hemorrhagic cases during an epidemic depends on the virus strain [2, 3].

It is considered an endemic disease in 112 countries in Africa, the Americas, the Eastern Mediterranean, Southeast Asia, and the Western Pacific. The World Health Organization (WHO) estimates that 40% of the world population, approximately 2.5 billion people living in tropical and subtropical areas, are at risk [4–6].

Dengue virus infection ranges from asymptomatic or subclinical infection to undifferentiated fever (viral syndrome), dengue fever or dengue hemorrhagic fever (DHF), including dengue shock syndrome (DSS) [7]; depending on the primary and secondary immune status of the host, its age, and infecting genotype [8].

After an average intrinsic incubation period of 4–6 days (ranges from 3–14 days), various nonspecific, constitutional symptoms and headache, backache, and general malaise may develop. Typically, the onset of dengue fever is sudden, with a sharp temperature rise and is frequently associated with a flushed face and headache. After that, there may be retro-orbital pain in the eye movement or eye pressure, photophobia, backache, and pain in the muscles and joint-bones [7].

Dengue symptoms range from fever, headache, arthralgias, myalgias, and maculopapular pruritic rash to severe hemorrhage, shock, and death. Cutaneous manifestations such as rash or petechiae are observed in 50 to 80% of cases around days 4 to 7 [9].

After the incubation period, the disease consists of 3 phases: the febrile, critical, and recovery phase [10]. The febrile phase extends from the second day after contact with the vector agent until the seventh day and is associated with facial flushing, skin erythema, generalized arthralgia, and myalgia. Anorexia, nausea, vomiting, hepatomegaly, progressive leukopenia, mucosal bleeding, and petechiae may also be found [11, 12].

After the resolution of high fever, a small percentage of patients progress to the critical phase, which lasts approximately 24 to 48 hours. This is characterized by extravasation of plasma with or without obvious bleeding. There is increased capillary permeability accompanied by increased hematocrit [13].

Atypical manifestations compromise the heart, liver, kidney, and nervous system [7]. Gastrointestinal manifestations include diarrhea, nausea, vomiting, and abdominal pain [14, 15].

According to WHO 2009, the classification criteria of dengue virus infection are living or traveling to a dengue-endemic area; having fever and two of the following: nausea, rash, aches and pains, tourniquet positive, leukopenia; and any warning signs (abdominal pain, persisting vomiting, clinical fluid accumulation, mucosal bleed, lethargy, restlessness, liver enlargement >2 cm, and increase in hematocrit concurrent with the rapid decrease in the platelet count) [16].

Acute pancreatitis is a rare manifestation of the virus that causes dengue; few cases have been described in the literature [17]. It usually occurs during the critical phase of dengue hemorrhagic fever, mainly during the third to seventh day from the onset of the fever [18, 19].

Diagnosis of acute pancreatitis requires the presence of at least two of the following three criteria: acute onset of persistent, severe epigastric pain often radiating to the back, elevation in serum lipase or amylase to three times or greater than the upper limit of normal, and characteristic findings of acute pancreatitis on imaging (contrast-enhanced computed tomography, magnetic resonance imaging, or transabdominal ultrasonography) [20].

During the acute phase, dengue virus antigens can be detected using ELISA or real-time polymerase chain reaction (PCR), while during the recovery phase, diagnosis can be addressed by detection of immunoglobulin (Ig) *M* using ELISA [21].

Infectious agents related to acute pancreatitis include viruses, bacteria, mycobacteria, parasites, and fungi, with a predominance of viruses in 65.3% [22].

Of the viral causes, dengue virus infection has been reported in 17 cases in the literature around the world, 13 from Asia, two from Latin America, and one from Europe [23].

Good clinical judgment is necessary for a patient with suspected acute abdomen due to pancreatitis since it can often lead to wrong decisions when the infection's cause is not considered in the differential diagnosis.

## 2. Case Presentation

A 26-year-old man with a past medical history of unspecified hepatomegaly at birth, and alcohol use disorder since he was 20 years of age, went to the Emergency Department complaining of 5 days of throbbing headache, arthralgias, myalgias, and a documented fever (40.4°C) that partially improves with ibuprofen and with no response to paracetamol.

The clinical course was exacerbated on day 2 of the initiation of symptoms by crampy (colicky) abdominal pain in the epigastrium with radiation to the right upper quadrant, accompanied by nausea and vomiting.

Admission physical exam revealed blood pressure (BP) of 120/90 mmHg, heart rate (HR) of 56 bpm, respiratory rate (RR) of 20 rpm, oxygen saturation of 97% in room air, and temperature of 36.6°C. Disseminated petechiae were observed on the patient's face, trunk, and extremities. Cardiac and lung exams revealed normal heart sounds, without rhythm alterations, no murmurs, and lung fields were clear, without wheezing, rhonchi, rales, or crepitus. Examination of the abdomen revealed scattered petechiae, increased abdominal perimeter (95 centimetres), tenderness to palpation in the epigastrium and right upper quadrant, without hepatosplenomegaly, and no guarding or rebound tenderness. Bowel sounds were present. The pulses in the extremities were regular, and there was no edema.

## 3. Investigation

Laboratory tests (Table 1) performed on the patient's hospital admission revealed a hematocrit of 49%, platelet count of 23.000, C-reactive protein (CRP) of 29.2, glutamic oxaloacetic transaminase (GOT) of 747 U/L, glutamic pyruvic transaminase (GPT) of 509 U/L, amylase of 229 U/L, lipase of 258 U/L, gamma-glutamyl transferase (GGT) of 604 U/L, alkaline phosphatase (ALP) of 208 U/L, lactate dehydrogenase (LDH) of 995 U/L, and lactic acid of 3.4 mmol/l, triglycerides of 267 mg/dl, cholesterol of 158 mg/dl, mild hypophosphatemia of 2.4 mg/dl, and albumin of 3.7 g/dl and glucose of 112 mg/dl.

Ultrasonography of the abdomen revealed an enlarged liver, with increased diffuse echogenicity and reinforcement of the periportal vessels, homogeneous structure, and fluid at the level of the left hepatic border that extended to Morrison's space. Gallbladder with walls of normal thickness, without endoluminal images, nondilated bile ducts, slightly hypoechoic pancreas, preserved shape and structure, and scant perisplenic fluid, slight bilateral pleural effusion associated with a consolidation process with air bronchogram was also seen.

Having ruled out the biliary cause of pancreatitis and searching for other causes, there were no calcium abnormalities, hypertriglyceridemia, endocrine abnormalities, or use of drugs associated with pancreatitis. Due to a history of fever and being a resident of an endemic area of the dengue virus, a viral panel was requested, resulting in positive IgM and IgG antibodies for dengue, with negative NS1 antigen (Table 2), qualifying it as dengue with warning signs.

## 4. Treatment and Outcomes

The patient was treated with intravenous antipyretics and fluid therapy.

The fever subsided two days after admission, and the patient maintained euthermic during the rest of his hospitalization.

Initially, the patient was placed on conservative treatment with nil by mouth, receiving nutritional maintenance with peripheral parenteral support based on 10% amino acids, 5% dextrose, 20% lipids, and B and C vitamins, with a caloric intake of 650 cal.

**Figure 1:**
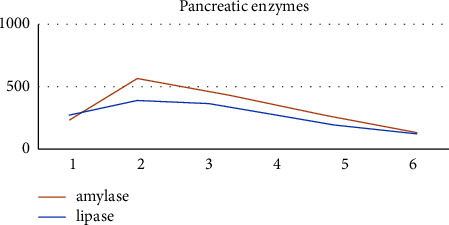
Pancreatic enzyme curve.

Abdominal pain almost completely disappeared on the first day of fasting. Parenteral support was maintained until day 5 of fasting when oral intake was reestablished. The patient began receiving oral tolerance with liquids and progressed to semisolid food and a low-fat diet; as abdominal pain remained absent and pancreatic enzymes diminished (Figure 1 and Table 3).

## 5. Discussion

According to the diagnostic criteria of WHO SEARO 2011, this case is defined as dengue hemorrhagic fever supported by clinical manifestations such as acute onset fever, high and continuous, lasting between two to seven days, in the presence of petechiae, in addition to thrombocytopenia (100.000 cells per mm^3^ or less) and hemoconcentration. The presence of liver enlargement is suggestive of DHF before the onset of plasma leakage. The presence of pleural effusion (chest X-ray or ultrasound) is the most objective evidence of plasma leakage, while hypoalbuminemia provides supporting evidence [7].

Literature mentions that abdominal pain is a common symptom (40%) in dengue infections and is more commonly associated with DHF. Diverse reasons in patients with DF could cause abdominal pain in the epigastrium. However, the diagnosis became apparent after having evidenced a significant elevation in pancreatic enzymes and discarding surgical cases by imaging. DF by itself can cause the already mentioned alterations on the hemogram, but hemoconcentration and marked thrombocytopenia, added to evidence of plasma leakage, classify the patients as hemorrhagic fever, having a greater opportunity of presenting with complications such as acute pancreatitis [24].

Furthermore, it is compatible with acute pancreatitis criteria. The crampy abdominal pain in the epigastrium was the symptom for searching medical consultation. It was accompanied by three times the elevation in pancreatic enzymes plus the finding of the hypoechoic pancreas on ultrasonography, a described characteristic of acute pancreatitis in relation to normal parenchyma [25].

In patients with characteristic abdominal pain and elevation in serum lipase or amylase to three times or greater than the upper limit of normal, no imaging is required to establish the diagnosis of acute pancreatitis [26].

Acute pancreatitis often occurs as a diffuse and generalized increased size of the pancreas, along with more imprecise limits and contours and decreased organ echogenicity. In the edematous form, the parenchyma is uniform and homogeneous, although hypoechoic with respect to normal parenchyma. In diffuse pancreatitis, the pancreas is hypoechoic and enlarged relative to the normal liver. As the inflammation becomes more severe, the decrease in echogenicity and the increase in size due to the higher liquid content in the interstitium becomes more evident [25].

The enlarged pancreas was found in 41 patients (29%), 10 (14%) of whom had mild DHF and 31 (44%) of whom had severe DHF in the Setiawan et al. study. The pancreas was hyperechoic relative to the liver in 36 patients (25%), isoechoic in 98 (69%), and hypoechoic in 8 (6%). It was concluded that most patients with DHF and epigastric pain do not have the enlarged pancreas, different echogenicity of the pancreas compared with the liver, or a dilated pancreatic duct [27].

Although dengue virus infection may share gastrointestinal signs and symptoms with acute pancreatitis, the differentiating factor in this case is the presence of elevated pancreatic enzymes, which do not occur in dengue fever alone, accompanied by the ultrasound abnormalities already described, which directs our diagnosis to acute pancreatitis associated with dengue virus infection, given the positivity for dengue IgM at the initial evaluation of the patient.

Acute pancreatitis is a common pathology presenting in the emergency room. Ecuador being an endemic country in dengue, it should be considered in the differential diagnosis.

Although abdominal pain or severe gastrointestinal involvement is a recognized feature during the critical phase of DHF (from the 3rd to 7th day from the onset of fever). In our case, gastrointestinal manifestations appeared on the second day of the onset [28].

The patient's age demographic is similar to the study by Eldigail et al., in which 51.7% of the patients were young (less than 40 years), and 46.3% belonged to the adult population [29]. In another case report, Ghweil et al. mention that the age of the patients is an average of 40.34 years ±15.74 [8].

A study carried out by Anjalie shows a slight predominance of the male gender, with a male : female ratio of 1.33 : 1.22 [30], which could be in agreement with our case.

The clinical presentation of acute pancreatitis in the patient we discussed was on the sixth day of disease evolution, consistent with several case reports in which acute pancreatitis was considered an atypical manifestation present within the first days of infection by dengue virus [31–33]. In contrast to the Ghweil et al. case report, this complication was reported three months after clinical recovery, as well as cases described in Egypt as the most common late complication [8].

In the case we illustrate, the most remarkable features were fever and myalgias/arthralgias. This is similar to the Ghweil case report, in which arthralgia, retroocular pain, and nausea were detected in 95, 92, and 52% of the cases, respectively. Likewise, in our case, abdominal pain and vomiting were present in the patient; data contrast with these manifestations in 26% of cases [8].

In a retrospective study of 8,559 patients with dengue fever, 67% showed abdominal and gastrointestinal symptoms. The most common symptoms were nausea (52%), followed by abdominal pain (36%), and vomiting (29%) [1].

However, abnormalities in liver function tests are reported in approximately 80% of cases [34, 35]. In our case report, the values are three times higher than the normal range. The data agrees with Souza et al., who found a higher frequency of elevation of the aspartate aminotransferase (AST) compared to the alanine aminotransferase (ALT) [36, 37].

In the current case, there were meaningful elevations on aminotransferases, which is characteristic of dengue with warning signs, even in the absence of abdominal pain or acute pancreatitis.

Shilpi reported in their study that no patients with dengue fever without warning signs develop raised transaminases or raised bilirubin. However, 15.9% of patients with dengue fever with warning signs develop hyperbilirubinemia with total bilirubin >mg/dl and without elevated liver enzymes [38].

Reported causes of abdominal pain in patients with dengue infection include hepatitis, pancreatitis, acalculous cholecystitis, gastritis, and peptic ulcer disease. Acute pancreatitis has been observed in 14% of patients with dengue hemorrhagic fever and abdominal pain. In one case series, all patients had mild acute pancreatitis. In the Khanna study, acute pancreatitis was reported in 14.5% of the causes of abdominal pain in patients with dengue fever [39].

Fever is not characteristic of acute pancreatitis, unless localized or systemic complications are present, attributing the fever presented in our case being provoked by the viral infection distinctive of dengue fever [26].

Although acute pancreatitis is an atypical manifestation of dengue, gastrointestinal manifestations were found in more than 70% of hospitalized patients in Veracruz, Mexico [1].

Laboratory tests performed on the patient revealed marked thrombocytopenia on admission, which correlates with what was described by Ramos, who published that patients with gastrointestinal manifestations had less than 100,000 platelets/mm^3^ [8].

The frequencies of anemia, thrombocytopenia, and leukopenia were similar to the studies by Ghweil et al., [8, 40, 41].

In addition to the symptoms suggestive of pancreatitis presented by our patient (abdominal pain and vomiting), the values of pancreatic enzymes were three times higher than the normal value. Pancreatic enzymes decreased around the sixth day; these data are similar to those described in the Kumar et al. case report in which serum amylase and lipase decreased after one week [42]. However, in the Anam et al. case report, the reported values of amylase and lipase were within normal parameters [43].

Commonly associated laboratory findings with DF include thrombocytopenia, leukopenia, hemoconcentration, and increased aminotransferases levels, as noted in the Shamim study [44]; nonetheless, increased pancreatic enzymes elevations are commonly present in the setting of acute pancreatitis.

Abdominal ultrasound is a valuable tool for detecting evidence of plasma leakage and diagnosing acute abdomen in DHF. The reported sonographic features include thickened gallbladder wall, ascites, splenomegaly, and pleural effusion [45]. Ultrasound findings on day one of admission can only detect ascites and not pancreatic edema, as shown in our patient, which did not report changes in the gallbladder or biliary tract. These data agree with the studies in the literature that show the normal pancreas in ultrasonography [46]. Jain's case report revealed the hypoechoic pancreas suggestive of pancreatitis and minimal ascites. It also showed serositis in the form of minimal bilateral pleural effusion, also seen in the chest computed tomography of our presented case [47].

Acute abdomen in DHF can be treated successfully by conservative measures [48].

Symptomatic treatment is required for pain, fever, and nausea/vomiting. Patients should be well hydrated (intravenously) and receive nil by mouth, as our patient was managed [49].

In most patients with acute pancreatitis, the disease is mild in severity, and patients recover in three to five days without complications or organ failure [26]. Data agrees with the presenting case in which abdominal pain had ceased by day 5 of fasting, and the patient began oral tolerance.

Finally, the serological diagnosis of dengue infection was proven by the IgM antibody and dengue NS1 antigen negative, which could be explained by the presentation time.

## 6. Conclusion

The differential diagnosis of an acute febrile syndrome with abdominal pain or gastrointestinal symptoms in patients in endemic areas or with a history of travel to certain regions considered endemic should include dengue.

Acute pancreatitis can even present as a late complication, so clinical suspicion should include this pathology in all patients with a history of clinical symptoms compatible with dengue.

## Figures and Tables

**Table 1:** Admission laboratory.

Hemogram		Reference values
Leukocytes	7.55	4.4–10 × 103 *μ*l
Neutrophils	5.19	2–8 × 103 *μ*l
Lymphocytes	1.59	1–4.4 × 103 *μ*l
Monocytes	0.61	0.08–0.88 × 103 *μ*l
Eosinophils	0.00	0.08–0.44 × 103 *μ*l
Hemoglobin	16.7	12.6–16.4 g/dl
Hematocrit	49.1	38–48%
MCV	85.8	76–96 fl
MCH	29.2	28–33 pg
Platelets	23,000	150–450
Distribution width	12	11.5–14.5%

Biochemistry		Reference values
Urea	20	16–48 mg/dl
Creatinine	0.90	0.50–1.30 mg/dl
Glucose	112	70–99 mg/dl
Sodium	1.40	133–145 mEq/l
Potassium	4.4	3.5–5.4 mEq/l
Chlorine	102	95–105 mEq/l
Magnesium	2.06	1.58–2.55 mg/dl
Calcium	8.4	8.4–10.2 mg/dl
Phosphorum	2.4	2.7-4-5 mg/dl
RCP	29.2	28–33 pg
Lactate dehydrogenase	995	135–250 U/L
Lactate	3.4	0.4–2.2 mmol/l

Hepatogram and pancreatic enzymes		Reference values
GOT	747	0–40 U/L
GPT	509	0–41 U/L
GGT	604	8–61 U/L
ALP	208	40–129 U/L
Total bilirubin	1.09	0.00–1.20 mg/dl
Direct bilirubin	0.84	0.00–0.30 mg/dl
Indirect bilirubin	0.25	0.00–0.70 mg/dl
Albumin	3.7	3.5–5.2 g/dl
PT/INR	12.01/1.01	11–14 sec
PTT	46.5	25–45 sec
Lipase	258	0–60 U/L
Amylase	229	28–100 U/L
Cholesterol	158	0–200 mg/dl
Triglycerides	267	0–200 mg/dl

**Table 2:** Infectious viral profile.

Viral profile	
NS1 dengue Ag	Negative
IgM dengue	Positive
IgG dengue	Positive
IgM cytomegalovirus	Negative
IgG cytomegalovirus	Positive
IgM Epstein Barr	Negative
IgG Epstein Barr	Positive
Hepatitis B (HBSAg)	No reactive
Hepatitis C	No reactive
Sars Cov2 (swab)	Negative

**Table 3:** Pancreatic enzyme levels.

Pancreatic enzymes	Day 1	Day 2	Day 3	Day 4	Day 5	Day 6
Amylase	229	389	357	250	170	122
Lipase	258	559	467	342	222	129

## Data Availability

The data used to support this study are included within the article.
